# Which Matters More in Fighting COVID-19—Government Policy or Community Participation?

**DOI:** 10.3389/fpubh.2022.927553

**Published:** 2022-07-12

**Authors:** Ying Qian, Jiaoling Huang, Laijun Zhao, Io Hong Cheong, Siqi Cao, Li Xiong, Qin Zhu

**Affiliations:** ^1^Business School, University of Shanghai for Science and Technology, Shanghai, China; ^2^School of Public Health, Shanghai Jiao Tong University School of Medicine, Shanghai, China; ^3^School of Management, Shanghai University, Shanghai, China; ^4^School of Social Development and Public Policy, Fudan University, Shanghai, China

**Keywords:** lockdown, COVID-19, system dynamics, SEIR model, community participation

## Abstract

**Objective:**

As a heavily populated megacity, Shanghai faces major epidemic risks. However, Shanghai's control of COVID-19 has been successful owing to both the strict government policy and wide community participation. Here, we investigated the impact of these stakeholders and examined who played a major role across different epidemic stages.

**Design:**

We extended the classic susceptible-exposed-infectious-recovered (SEIR) model considering the heterogeneous contact structure in four social sceneries, i.e., school, workplace, public entertainment venues, and neighborhood community, which could reflect the impact of lockdown policy and wide participation of residents happened at the community level.

**Result:**

The simulation results showed that without lockdown policy and only with community participation, the daily new confirmed cases would gradually increase to more than 7,000 [292/1,000,000] at the end of Sep. However, without community participation and only with a lockdown policy, the daily new confirmed cases sharply decreased to 30 [1.2/1,000,000] at the end of the 1st month and remained low for several months. However, when a lockdown policy was gradually lifted, the new confirmed cases increased exponentially, eventually reaching more than 17,000 [708/1,000,000]. Therefore, a government lockdown policy was necessary for the rapid control of COVID-19 during the outbreak stage while community participation is more important in keeping the number of new confirmed cases low during the reopening stage.

**Conclusion:**

Government lockdown policy and community participation play different roles in the control of COVID-19 at different stages of the epidemic: although the government played a leading role in setting up policies, the broader participation of community fever clinics (CFCs) and the general public were especially crucial in winning the battle against COVID-19 in the long run.

## Introduction

China experienced the outbreak of COVID-19 in January 2020 that started in Wuhan and then spread to other cities. With 24–28 million permanent residents and a high population density, Shanghai was considered at high risk for epidemic spread (1). However, Shanghai performed surprisingly well-in terms of controlling the spread of COVID-19. In the first month of the epidemic (from 20 January to 19 February 2020), Shanghai confirmed a total of 346 new daily confirmed cases, of which around 255 were infected locally. The locally infected new confirmed cases peaked at 24 on 2 February 2020 and then gradually decreased, approaching zero at the end of the first month of the epidemic. In the following 8 months (20 February−19 October 2020), only 10 new confirmed cases were infected locally (2).

**Figure 1:**
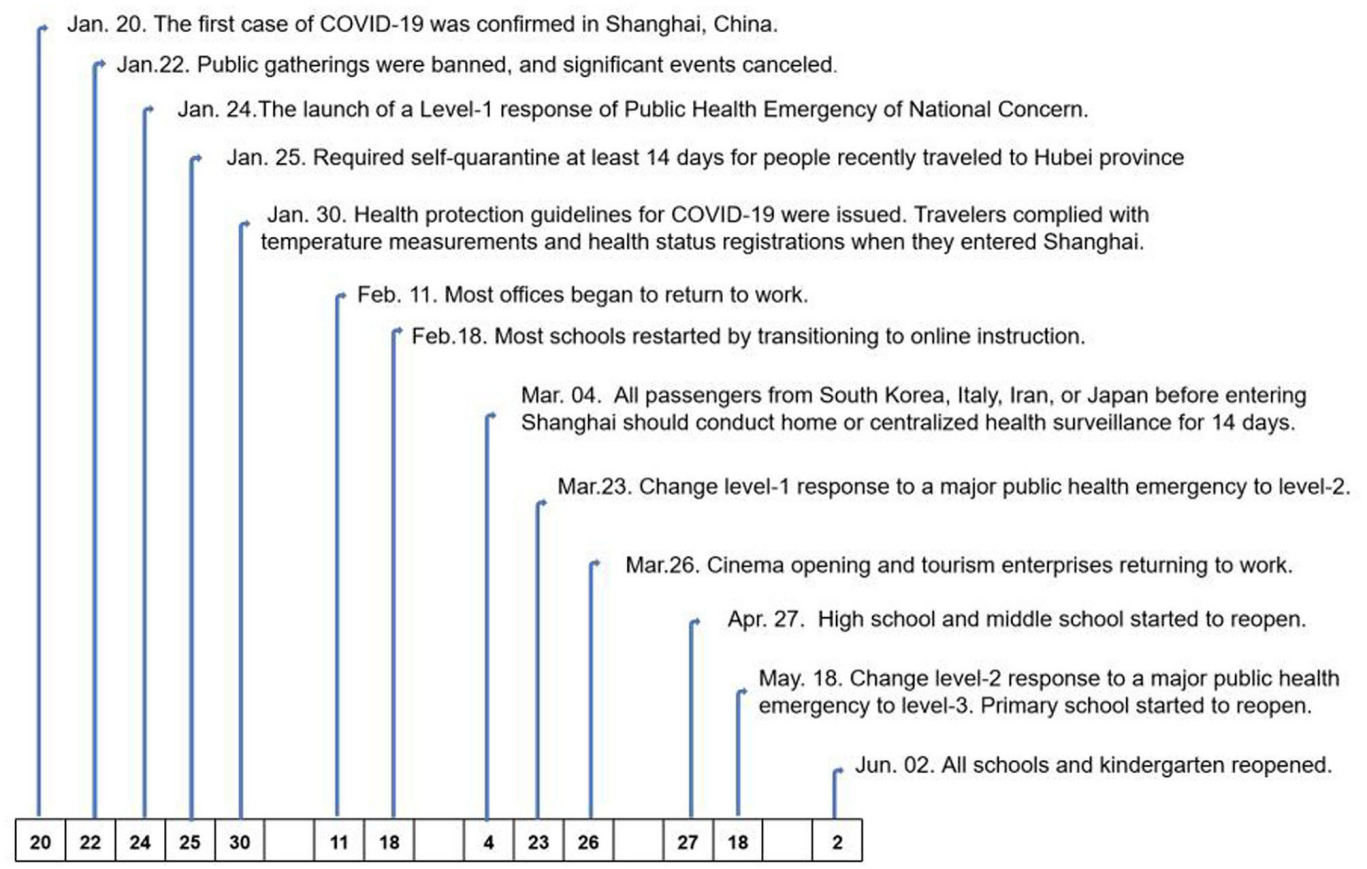
Lockdown and reopening timeline.

Many factors helped to prevent and control COVID-19 in Shanghai. At the government level, a series of policies were issued soon after the identification of the first confirmed case of COVID-19 in Shanghai. A workplace lockdown and a ban on public gatherings were implemented; these policies were not reversed until 11 February 2020. All schools were closed in March 2020 and then gradually reopened from 26 April 2020. Universities remained closed until the fall semester, and all courses were delivered online until the universities reopened (3). Public entertainment venues, such as cinemas, theaters, Internet cafes, and gyms, were also closed (see Figure 1). A quarantine of people arriving in Shanghai was another important policy implemented by the government (4). The lockdown and reopening timelines are displayed in Figure 1.^1^

At the same time, a great deal has been done at the community level to combat COVID-19 in Shanghai (5, 6). The concept of community participation in health was formally articulated by the World Health Organization (WHO) at Alma Ata in 1978 to achieve the “Health for All” strategy, acknowledging that primary care is a key component of local involvement in community participation (7). Community health service centers (CHSCs) are the primary healthcare institutions in urban China. CHSC general practitioners (GPs) conducted daily checks of the physical well-being of residents who were self-isolating at home. In addition, community fever clinics (CFCs) were established in CHSCs after the outbreak of COVID-19, which helped with the prompt identification of patients with suspected fever symptoms, the rapid isolation of suspected patients, and the transfer of patients to nearby large general hospitals with greater diagnosis and treatment capabilities. GPs and neighborhood committees, and residents actively participated in fighting COVID-19, such as through epidemiological investigation and vaccination mobilization (8). Moreover, residents voluntarily changed their behavior, such as reducing travel plans for the Spring Festival, wearing masks when going out, having online meetings and communication, using online shopping, staying home rather than gathering, and socializing in the community even for the elderly (9, 10).

In sum, the effective control of the COVID-19 epidemic in Shanghai was possible because of all the abovementioned efforts from both government policies (11). and community participation of CFCs, Neighborhood Committee, and residents (12–14). However, as most existing studies analyzing the prevention and control intervention measures implemented in China have used qualitative methods, describing what has been done, at what time, and with what impact (5, 15, 16), several questions need to be further addressed, such as What is the impact of each stakeholder on controlling COVID-19? Who played a major role at each stage of the epidemic? Is there a shift of dominant role across different stages of the epidemic? In the present study, we used a modeling approach to quantitatively investigate the impact of these stakeholders on the control of COVID-19.

## Materials and Methods

### Model Construction

**Figure 2:**
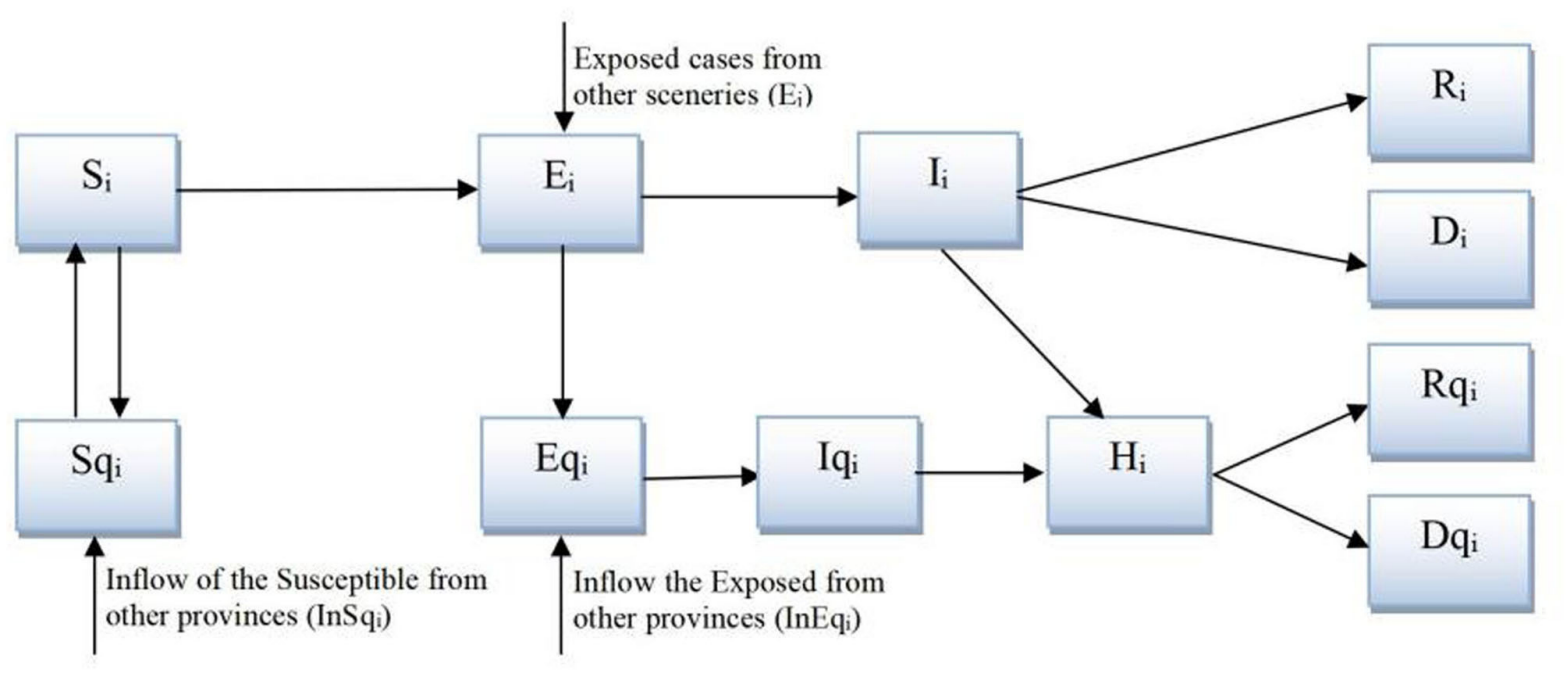
The extended susceptible-exposed-infectious-recovered (SEIR) model.

We developed a system dynamics model based on the classic susceptible-exposed-infectious-recovered (SEIR) model (17), which reflects the spread of a virus through contact and transmission between four groups of individuals: *S* (the susceptible), *E* (the exposed, which refers to the infected population without symptoms, especially during the incubation period), *I* (the infected, which refers to the infected population with symptoms), and *R* (the removed that includes the recovered and died population). Several extensions to this model are made, as shown in Figure 2. First, to comply with the track, trace, and quarantine policy in Shanghai, we extended the model to include *Sq, Eq, Iq*, and *Rq*, representing the quarantined groups of *S, E, I*, and *R*. Second and more importantly, we further disaggregated the model into four social scenarios—schools, workplaces, public entertainment venues, and neighborhood communities (18), within which the contact rate changed according to the mandatory lockdown policy issued by the government for the first three and according to voluntary behavior change in the general public for the last.

### Model Structure

As the quarantined and hospitalized population cannot contact other people, the total population contacting others in each scenery, *N*_*i*_, can be represented as *N*_*i*_ = *S*_*i*_ + *E*_*i*_ + *I*_*i*_ + *R*_*i*_ + *Rq*_*i*_. The transmission of the COVID-19 happens when *S* contacts *E* or *I*. However, not all people are contagious during the incubation period. Suppose θ percent of E are infectious. All *I* are contagious, but many are isolated at CFCs. Suppose μ percent of *I* are kept in isolation. The only source of infection is then θE + (1 – μ) × I. Given that *c* as the contact rate and β as the infectivity, the transmission of S to E is (θEi + (1 – μ) × I) × (S/N) × βc. The track, trace, and quarantine approach can only trace close contacts of confirmed cases. With q percent of infected people being quarantined, the transmission of E to Eq is (1 – μ) × I × (S/N) × βcq, and the transmission of S to Sq is (1 – μ) × I × (S/N) × (1 – β) × cq.

Therefore, the model can be represented as follows:


(1) $$\begin{array}{l} S_{i}=S_{i0}+\int_{0}^{t}(Sq_{i}/T1-((1-\mu )I_{i}+\theta E_{i})\beta c_{i}S_{i}/N_{i}\\ -(1-\mu )I_{i}(1-\beta )c_{i}qS_{i}/N_{i})dt \end{array}$$

(2) $$\begin{array}{l} Sq_{i}=Sq_{i0}+\int_{0}^{t}((1-\mu )I_{i}(1-\beta )c_{i}qS_{i}/N_{i}\\ -Sq_{i}/T1+InSq_{i})dt \end{array}$$

where T1 is the duration of quarantine;


(3) $$\begin{array}{l} E_{i}=E_{i0}+\int_{0}^{t}((1-\mu )I_{i}(1-\beta )c_{i}(1-q)S_{i}/N_{i}\\ +\theta E_{i}\beta c_{i}S_{i}/N_{i}-E_{i}/T2+E_{j})dt \end{array}$$

(4) $$\begin{array}{l} Eq_{i}=Eq_{i0}+\int_{0}^{t}((1-\mu )I_{i}(1-\beta )c_{i}qS_{i}/N_{i}-Eq_{i}/T2\\ +InEq_{i})dt \end{array}$$

where T2 is the average incubation period;


(5) $$\begin{array}{c} I_{i}=I_{i0}+\int_{0}^{t}(E_{i}/T2-I_{i}/T3-\alpha_{I}I_{i}-\gamma_{I}I_{i})dt \end{array}$$

(6) $$\begin{array}{c} Iq_{i}=Iq_{i0}+\int_{0}^{t}(Eq_{i}/T2-Iq_{i}/T3)dt \end{array}$$

(7) $$\begin{array}{c} H_{i}=H_{i0}+\int_{0}^{t}(I_{i}/T3+Iq_{i}/T3-\alpha_{H}H_{i}-\gamma_{H}H_{i})dt \end{array}$$

where T3 is the waiting time to be admitted to the hospital, which is affected by testing capacity and hospital facility availability;


(8) $$\begin{array}{c} R_{i}=R_{i0}+\int_{0}^{t}(\gamma_{I}I_{i})dt \end{array}$$

(9) $$\begin{array}{c} D_{i}=D_{i0}+\int_{0}^{t}(\alpha_{I}I_{i})dt \end{array}$$

(10) $$\begin{array}{c} Rq_{i}=Rq_{i}+\int_{0}^{t}(\gamma_{H}H_{i})dt \end{array}$$

(11) $$\begin{array}{c} Dq_{i}=Dq_{i}+\int_{0}^{t}(\alpha_{H}H_{i})dt \end{array}$$

where γ_*I*_ and γ_*H*_ represent the recovery rates of I and H, respectively, and α_*I*_ and α_*H*_ represent the death rates of I and H, respectively.

The parameter settings were in line with previous literature (19–21), and the detailed information is presented in Appendix 1.

### Model Validation

**Figure 3:**
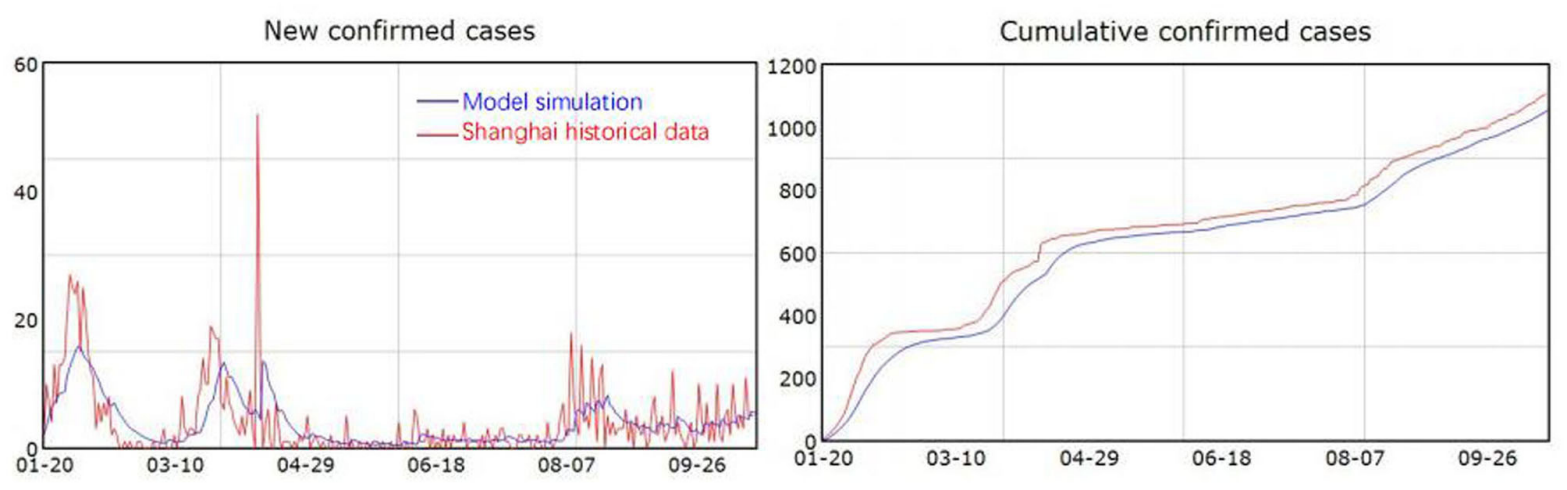
Simulation results and historical data.

The model was constructed in Vensim 8.0.9. The model simulates the progression of COVID-19 in Shanghai over the 9-month period from 20 January 2020, when the first case of COVID-19 appeared in Shanghai, to 19 October 2020, long after the COVID-19 outbreak in Shanghai had declined. The SEIR model has been widely used for investigating COVID-19 (17, 18, 22), suggesting that the model structure is valid. The model parameter setting is mostly referred to previous literature (19–21). We compared the model simulation results with historical data published daily on the website of the Health Commission of Shanghai. As shown in Figure 3, the simulated new confirmed cases and cumulative confirmed cases fit well-with the historical data, which increases confidence in the model.

## Results

Using the model, we focused our investigation on the effectiveness of the lockdown policy and community participation in the fight against COVID-19 in Shanghai. The lockdown policy mainly changes the contact rate in schools, workplaces, and public entertainment venues. Community participation mainly includes residents' behavior changes in terms of reducing the contact rate in the neighborhood community and the enhancement of CFCs to cut the chain of infection as early as possible.

### Scenario 1: The Effectiveness of Lockdown Policies

Lockdown policies are included in the model through changes in the contact rate in different scenarios, as shown in Table 1.

**Table 1:** Heterogeneous contact rate under lockdown policies.

**Contact rate setting**	**Explanation**
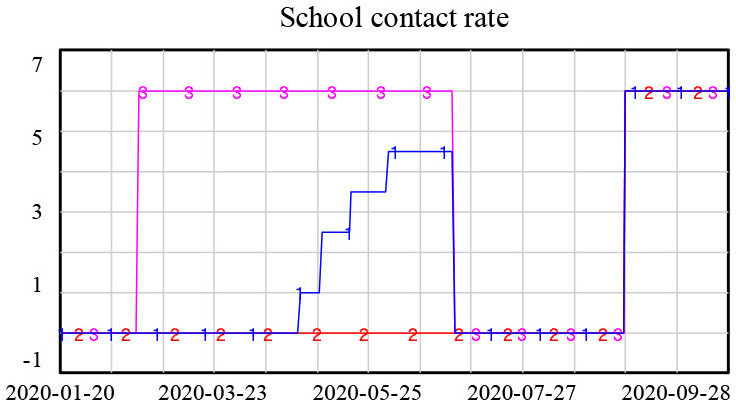	**1. Base (Actual):** school contact rate remained 0 until April 27th and gradually increased. After summer holiday, it reached normal level. **2. Extended lockdown:** school contact rate remained 0 until Sep and then it returned normal level. **3. No lockdown:** school contact rate was 0 for winter and summer holiday, otherwise, it remained normal level.
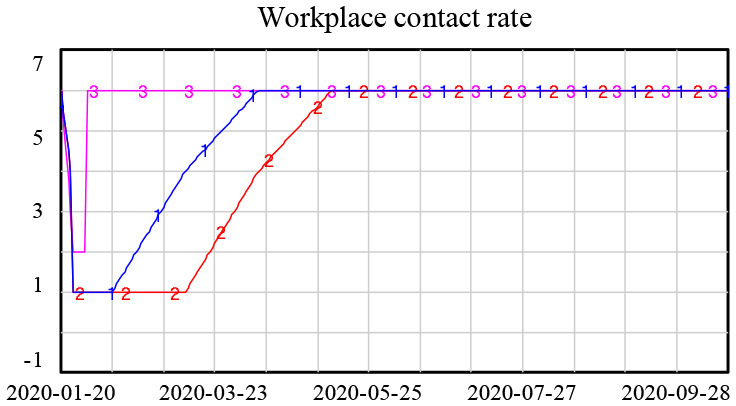	**1. Base (Actual):** Workplace contact rate reduced to low level until February 11th and gradually returned to normal rate in 2-month time. **2. Extended lockdown:** workplace remained lockdown for one more month compared to Base scenario. **3. No lockdown:** workplace contact rate reduced to low level during Spring Festival and returned to normal soon afterward.
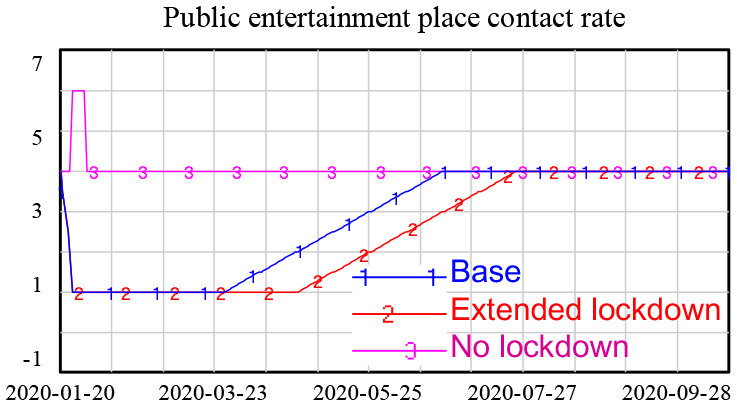	**1. Base (Actual):** public place contact rate reduced at low level until Feb 11 and gradually returned to normal rate in 3-month time. **2. Extended lockdown:** public place remained lockdown for one more month compared to Base scenario. **3. No lockdown:** public place contact rate increased during Spring Festival and returned to normal soon afterward.

**Figure 4:**
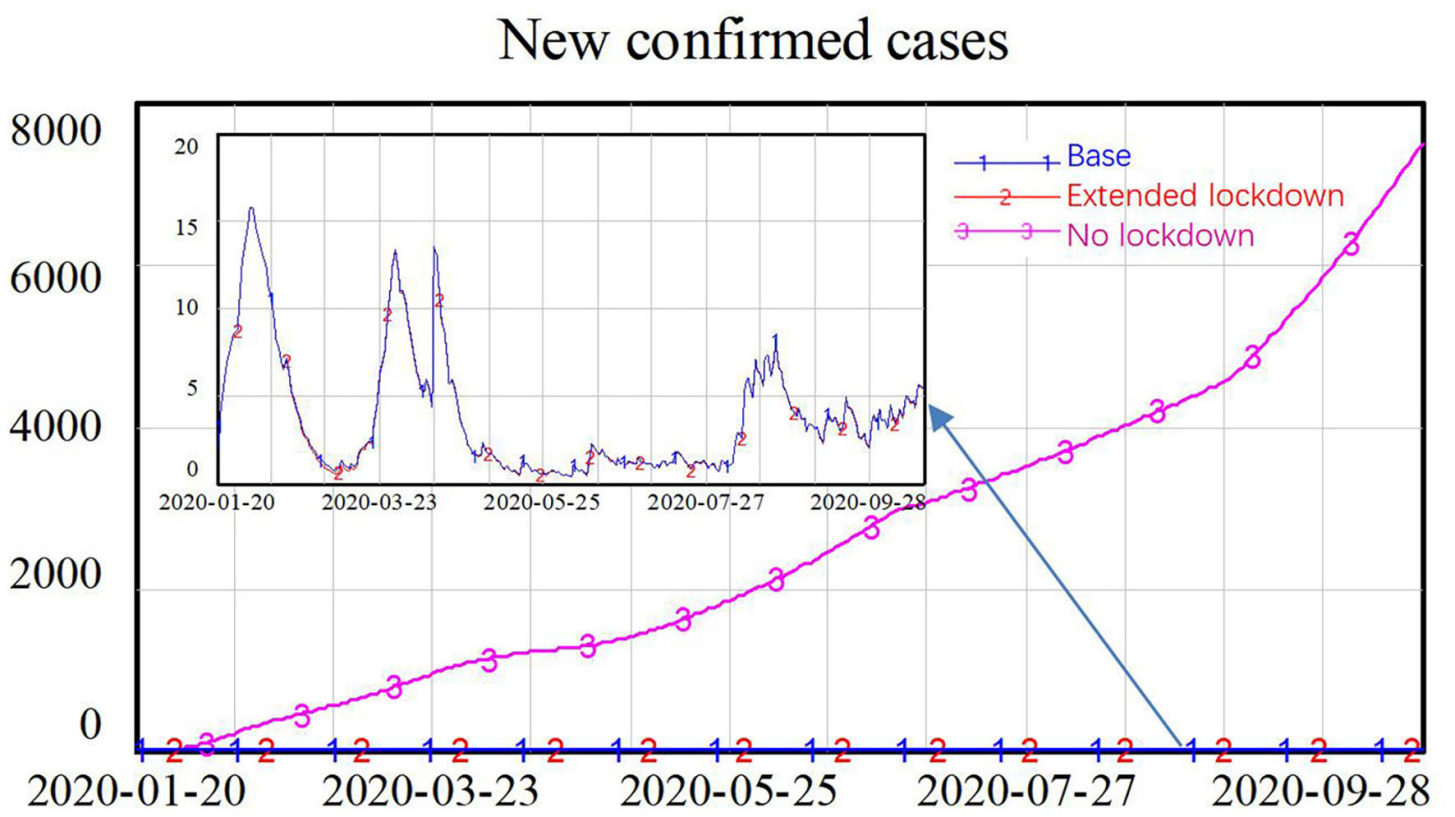
Simulation results for different lockdown policies.

The simulation results showed that the lockdown policy is effective in controlling the spread of COVID-19. Without a lockdown policy, the number of new confirmed cases gradually increased over time, approaching 8,000 new confirmed cases at the end of the simulation. However, a long period of lockdown is not necessary. After the number of new confirmed cases reached a low level in February, an extended lockdown had little impact on future numbers of new infections, as shown in Figure 4.

### Scenario 2: The Effectiveness of CFCs

After the outbreak of COVID-19, CFCs were additionally established to help identify and isolate suspected patients at the community level and to transfer these patients to nearby large general hospitals for further treatment. We assumed that the fever clinics could isolate 60% of the infected population under normal conditions, and we increased this by 40 percentage points during the COVID-19 epidemic, reaching 100% isolation of the infected population with symptoms. We also simulated another three scenarios assuming the fever clinics could only isolate 40, 20, and 0% of the infected population under normal conditions, yielding estimates of 80, 60, and 40% isolation rates during the COVID-19 pandemic, as shown in Table 2.

**Table 2:** Parameter setting for isolation of the infected population with symptoms at community fever clinics (CFCs).

CFC 100	Effectiveness of fever clinics = 0.6+step (0.4, 13)
CFC 80	Effectiveness of fever clinics = 0.4+step (0.4, 13)
CFC 60	Effectiveness of fever clinics = 0.2+step (0.4, 13)
CFC 40	Effectiveness of fever clinics = step (0.4, 13)

**Figure 5:**
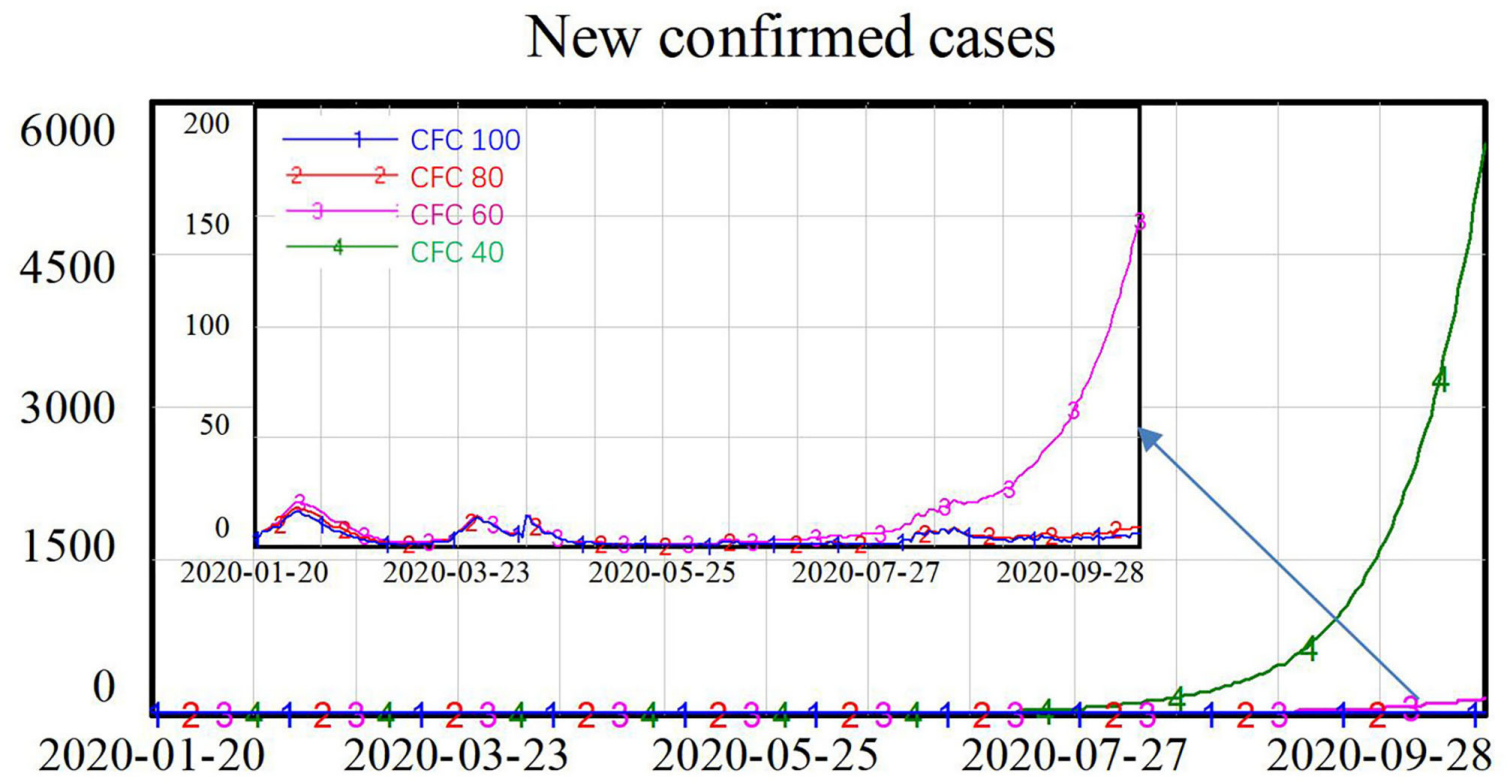
Simulation results for various effectiveness of community fever clinics (CFCs).

The CFCs play an important role in fighting COVID-19. As shown in Figure 5, when the percentage of the infected population with symptoms (I) being isolated is at the low level of 40%, the number of new confirmed cases increased exponentially, approaching 6,000 at the end of the simulation. When 60% of the infected population with symptoms could be isolated, the number of new confirmed cases were declined to around 150. When more than 80% of the infected population could be isolated by the CFCs, there were only around 10 new confirmed cases, and the spread of the virus was well-controlled.

### Scenario 3: The Effectiveness of Residents' Behavior Changes

**Figure 6:**
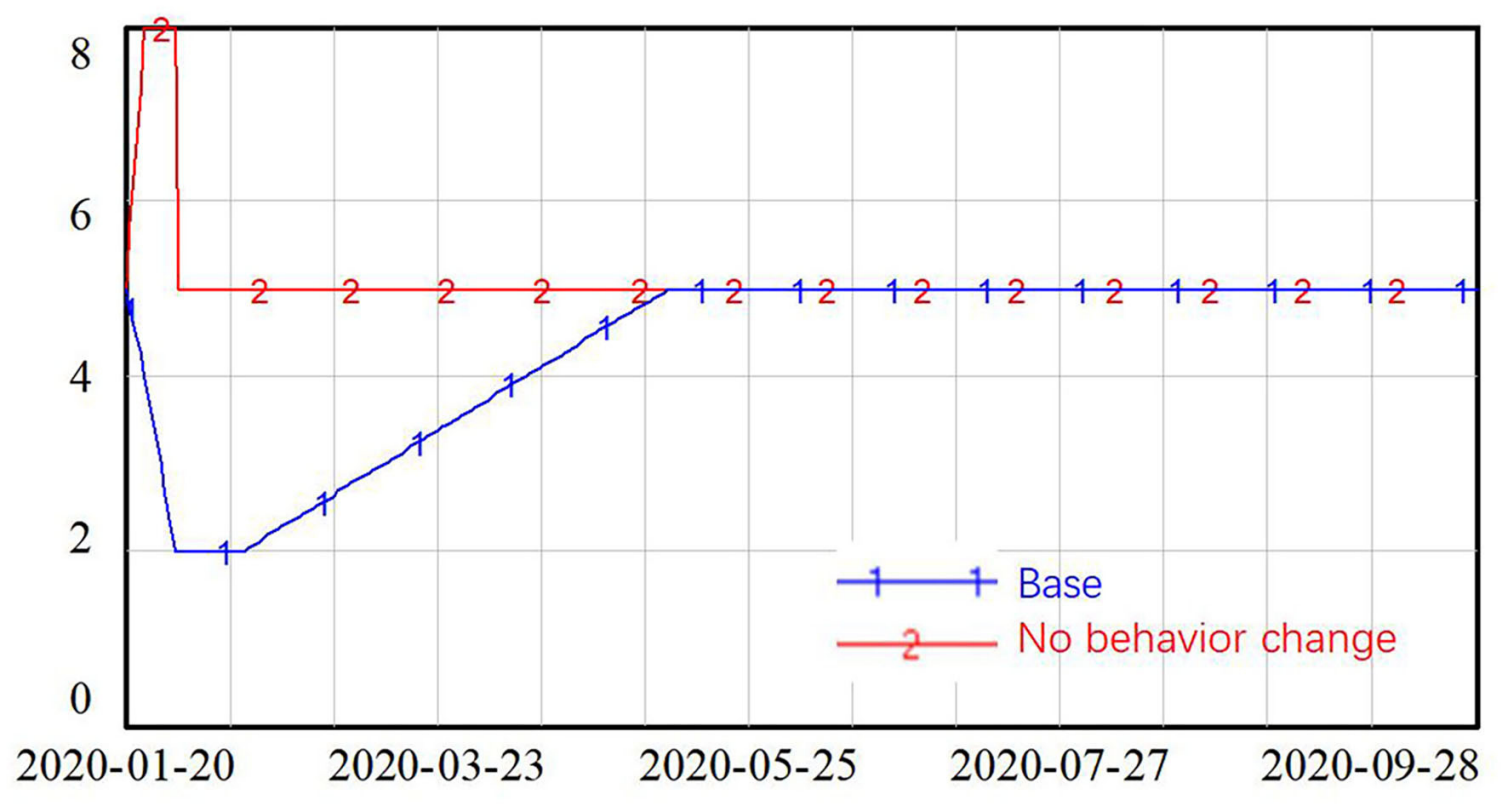
Simulation setting for contact rate at neighborhood community.

To help control the spread of the virus, residents in Shanghai changed their behaviors to reduce the contact rate in neighborhood communities. In the absence of the COVID-19 epidemic, people in Shanghai customarily participate in many social gatherings during the Spring Festival, reuniting with family members and friends. However, during the Spring Festival periods in 2020 and 2021, most people stayed at home and got in touch with their family members and friends online. Two situations were tested, which are as follows: the base scenario presents the real situation where people reduced their contact with others in January and February 2020 and then gradually increased to their normal level in May 2020. The other situation considered was that of no behavior change on the part of residents—here, the contact rate was set to increase by 60% during the Spring Festival and then decline back to the normal level after the holiday, as shown in Figure 6.

**Figure 7:**
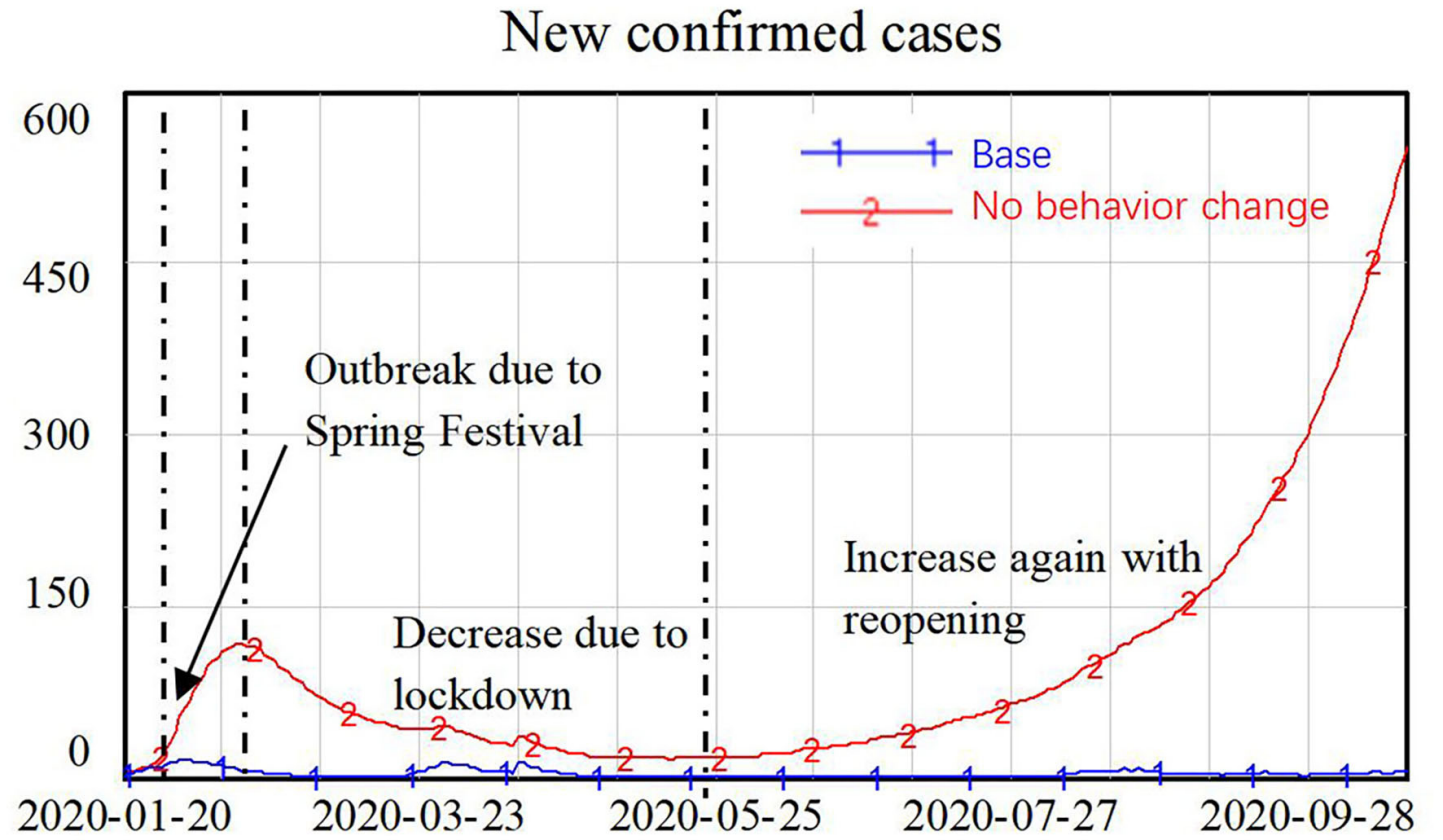
Simulation results with and without residents' cooperation in terms of behavior change.

The simulation results in Figure 7 shows that without behavior changes on the part of the general public, the number of new confirmed cases first peaked during the Spring Festival, when people were gathering with family members and friends. The number of new confirmed cases then declined after the Spring Festival, with the lockdown policy in place, but it then increased again with the reopening of the economy. Exponential growth in new confirmed cases was identified in this simulation, with the number of new confirmed cases reaching around 500 people at the end of the simulation.

### Scenario 4: Government Lockdown Policy vs. Community Participation

Besides the base scenario representing the actual situation that happened in Shanghai, other two scenarios were simulated: first, only government lockdown policies were implemented, without enhanced CFC intervention or behavior change of the general public; second, with only community participation, i.e., enhanced CFC intervention and resident's behavior change, but no government lockdown policy. The parameter setting is presented in Table 3.

**Table 3:** Scenario setting for only lockdown and only community participation.

**Scenarios**	**Contact rate**	**Contact rate**	**Contact rate**	**Contact rate at**	**Effectiveness**
	**at school**	**at workplace**	**at public venues**	**neighborhood**	**of CFC**
Base	Reduced	Reduced	Reduced	Reduced	0.6+ step (0.4, 13)
Only lockdown	Reduced as base	Reduced as base	Reduced as base	No behavior change	0.6
Only community participation	No change	No change	No change	Reduced as base	0.6+step (0.4, 13)

**Figure 8:**
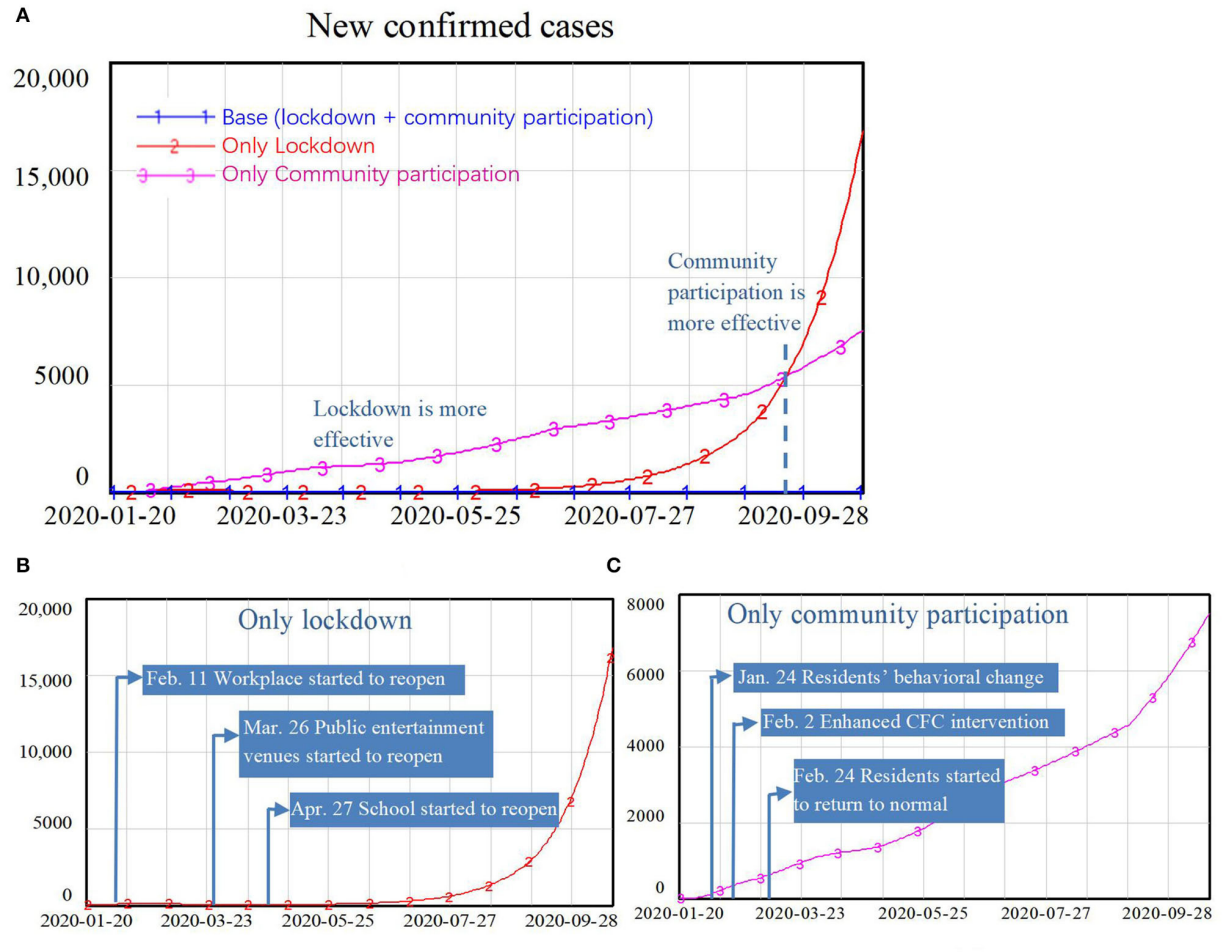
Simulation results comparing the effects of lockdown policy and community participation: part **(A)** compares the only lockdown scenario with only community participation scenario; part **(B)** represents the lockdown scenario only; and part **(C)** represents the community participation scenario only.

From the simulation results in Figure 8, we can see that with only the lockdown policy, the number of new confirmed cases declined to a very low level during the first several months of the COVID-19 epidemic as a result of reductions in the contact rates through schools, workplaces, and public entertainment venues. However, after the reopening, contact rates gradually returned to normal levels in the absence of community participation, in the long run, the number of new confirmed cases increased exponentially, reaching 17,000. In contrast, with only community participation and no lockdown policy, the number of new confirmed cases continued to increase slowly over time. Even when residents changed their behavior such that contact in neighborhood communities was reduced and the CFCs effectively identified and isolated 80% of infected patients, it was not possible to eliminate the virus because people in the incubation period could still spread the virus in schools, workplaces, and public entertainment venues. The number of new confirmed cases increased almost linearly, reaching around 7,500 at the end of the simulation.

## Discussion

Government lockdown policy and community participation have different impacts on the control of COVID-19 at different stages of the epidemic.

The outbreak stage will be controlled if a strict lockdown of workplace, schools, and public entertainment places have been implemented for 2 weeks, as most COVID-19 cases have an incubation period of <14 days and most people who were during the incubation period will develop symptoms in this lockdown period and then be isolated by a CFC or quarantined in a hospital (23, 24). This approach would reduce the sources of infection to a very low level and limit further infections, getting the epidemic under control in a short time frame. A longer lockdown will have little impact on the control of COVID-19 with the longer lockdown. Considering the major social and economic costs of lockdowns (25, 26), reopening at the earliest possible stage should be a preferred policy. However, reopening would increase the contact rate, which may lead to another increase in the number of new infections (24). Many countries have witnessed the second and third waves of the COVID-19. For example, a study of the first and second waves of the COVID-19 pandemic in Africa showed that a large portion of the African countries experienced a second wave after the loosening of public health and social measures, such as canceled public events, closed public transport, and international travel controls (27).

Facing this challenge, community participation is the key factor for keeping COVID-19 under control at this later stage. In Shanghai, CFCs are housed within CHSCs, which are the main primary care institutions. Shanghai was among the first cities to implement China's General Practice System—the key target of the country's new healthcare reform (28), and after the 10-year fundamental construction of the General Practice System, 225 CFCs were able to be established in a very short period in Shanghai. These CFCs were distributed near or right in the residential communities (29), thus CFCs can help to identify patients earlier once the suspected patients visited CHSC and to promptly isolate them at the community level to keep the spread of the virus as low as possible. CFCs treated patients with a fever in separate clinics, preliminary diagnosed suspected patients with COVID-19 through epidemiological investigation or acid-based diagnostic tests (some CFCs were able to perform such tests), and helped to transfer suspected patients to nearby hospitals. It is undeniable that CFCs played an important role in screening at-risk patients and cutting the infection chain after loosening the lockdown policies.

Besides the contribution of CFCs, neighborhood committees and residents' participation have also been critical for the control of the COVID-19 epidemic. The positive responses at the personal respective include wearing masks when going out, reducing visits and gatherings, and using the Internet for shopping and meetings (30, 31). People in Shanghai were also actively engaged in the anti-epidemic movement and even participated in community management as volunteers, such as measuring temperatures as gatekeepers at community entry points, assisting with the epidemiological investigation and information collection for inflow population from other provinces, and participating in vaccination promotion (32). All these activities directly reduced the contact rate and source of infection, which served to cut the chain of infection (33, 34).

## Conclusions

This research was built on a system dynamics model with a heterogeneous contact structure to investigate the impact of government policy, especially lockdown of school, workplace, and public entertainment venues, and the impact of community participation, especially CFC and residents' protective behavior, on the control of COVID-19. Simulation results illusted that without lockdown policy the daily new confirmed cases would gradually increase, reaching more than 7,000 [292/1,000,000] at the end of simulation. While without community participation, the daily new confirmed cases would sharply decrease in the first month but increase exponentially when the lockdown policy was lifted. This result implied that the broader participation of the community was especially crucial in winning the battle against COVID-19 in the long run, though the government lockdown policy played a dominant role in the outbreak stage of the epidemic. This result may not apply to places with different social mixes, such as rural areas. Further research on changing model structure and parameter setting is needed for places with the different social mixes. However, for most cities, with a large portion of the working population and many schools, wider participation from the community level for reduced contact and early identification and isolation of infected patients could help limit the spread of COVID-19 after the resume of normal life.

## Data Availability Statement

The original contributions presented in the study are included in the article/Supplementary Material, further inquiries can be directed to the corresponding author/s.

## Author Contributions

YQ and JH were involved in literature search, study design, data collection, model building, result analysis and interpretation, and writing. LZ, SC, and IC were involved in the literature search, result analysis and interpretation, and writing. LX and QZ were involved in literature search, data collection, and result analysis. All authors contributed to the article and approved the submitted version.

## Funding

The authors acknowledge the support from the Science and Technology Committee of Shanghai Municipality Soft Science Research Plans (Nos. 21692190200 and 22692192300), the National Social Science (major project 21ZDA105 and project 18AZD005), and the National Natural Science Foundation of China (Project No. 71874108).

## Conflict of Interest

The authors declare that the research was conducted in the absence of any commercial or financial relationships that could be construed as a potential conflict of interest.

## Publisher's Note

All claims expressed in this article are solely those of the authors and do not necessarily represent those of their affiliated organizations, or those of the publisher, the editors and the reviewers. Any product that may be evaluated in this article, or claim that may be made by its manufacturer, is not guaranteed or endorsed by the publisher.

## Footnotes

^1^All the detailed information is collected from the Weibo (microblog) account of the Information Office of Shanghai Municipality at https://weibo.com/u/2539961154. This account is used by city authorities for releasing official information. Updates of COVID-19 intervention and prevention policies were all published on this account.
