# Supplementary material for: Which Matters More in Fighting COVID-19—Government Policy or Community Participation?
Source: Front Public Health. 2022 Jul 12;10:927553. doi: 10.3389/fpubh.2022.927553 (PMC9315311; doi:10.3389/fpubh.2022.927553)
Supplement: Supplementary file 1 [file Table_1.DOCX]

# Appendix 1 Parameter setting of the system dynamics model

| **Parameter and definition** | **Value** | **Unit** | **Source and Explanation** |
| --- | --- | --- | --- |
| N_1_: School population in Shanghai | 2.08 M | Person | From the statistics of Shanghai |
| N_2_: Working population in Shanghai | 15.49 M | person | From the statistics of Shanghai |
| N_3_= N_4_=N: Total population in Shanghai | 24.01 M | Person | From the statistics of Shanghai |
| C_1_: Contact rate at school | 6 | times/day | According to the literature^1^ |
| C_2_: Contact rate at work | 6 | times/day | According to the literature^1^ |
| C_3_: Contact rate at public entertainment places | 4 | times/day | According to the literature^1^ |
| C_4_: Contact rate at community | 5 | times/day | According to the literature^1^ |
| C: Average of contact rate in Shanghai | 14 | times/day | Weighted average of four sceneries (N_1_*C_1_+N_2_*C_2_+ N_3_*C_3_+N_4_*C_4_)/N, which is in accordance with the literature^2^ |
| β: Probability of transmission | 0.035 | 1/times | R0=βc*T2=2.55^3^ |
| T2: The average incubation period | 5.2 | day | The mean incubation period is 5.2 days. ^3^ |
| Q: Quarantined rate of exposed individuals | 0 | % | Initialized as 0 and gradually increased to 0.9 as Level-1 emergency response was implemented.^4^ |
| T1: The duration of quarantine | 14 | day | The duration of the quarantine is 14 days in Shanghai^4^ |
| T3: The waiting time to be admitted to hospital | 1 | day | In Shanghai, infected people with symptoms are immediately accepted to hospital. |
| $\mu$: Effectiveness of fever clinics to isolate patients infected with COVID-19 | 0.6 | % | The initial value was 60% and after the implementation of specialized fever clinics it increased to 100%. |
| γ_I_: Recovery rate of individuals in I | 0.07 | 1/day | Individuals with a mild symptom or an asymptomatic infection recovered within 14 days on average. |
| γ_H_: Recovery rate of individuals in H | 0.01 | 1/day | Starting from 0.01 per day and increased as treatment of COVID-19 improved. |
| α_I,_α_H_: Death rate | 0.004 | 1/day | Starting from 0.004 per day and reduced as treatment of COVID-19 improved. |
| θ: Infectious weight in incubation period | 0.5 | % | Reported from news about COVID-19. |

Reference:

Prem K, Liu Y, Russell TW, et al. The effect of control strategies to reduce social mixing on outcomes of the COVID-19 epidemic in Wuhan, China: a modelling study. L*ancet Public Health* 2020; **5**: e261-e70.

Tang B, Wang X, Li Q, et al. Estimation of the Transmission Risk of the 2019-nCoV and Its Implication for Public Health Interventions. *J Clin Med.* 2020; ***2*:** 462.

Li Q, Guan X, Wu P, et al. Early Transmission Dynamics in Wuhan, China, of Novel Coronavirus–Infected Pneumonia. *New Engl J Med* 2020; **13**: 1199-207.

Zhao J, Jia J, Qian Y, et al. COVID-19 in Shanghai: IPC Policy Exploration in Support of Work Resumption Through System Dynamics Modeling. *Risk Manag Healthc P* 2020; **13**:1951-63.
